# Cadaveric training of total hepatic vascular exclusion for juxtahepatic venous injury: a technical study

**DOI:** 10.1007/s00423-026-04288-9

**Published:** 2026-09-28

**Authors:** Tuna Aras, Julia Khabyuk, Nele Bunsen, Tobias Hilje, Janina Fischer-Mertens, Frank Eifinger, Rabi Raj Datta, Payman Majd, Martin Dübbers, Margarethe Draga, Martin Scaal, Michael Thomas, Stefan Saad, Reza Vahdad, Grigore Cernaianu

**Affiliations:** 1 Department of Vascular Surgery, EVK Bergisch Gladbach, Ferrenbergstraße 24, 51465 Bergisch Gladbach, Germany; 2 Department of Anatomy, Faculty of Medicine, University of Cologne, Joseph-Stelzmann Str. 9, 50931 Köln, Germany; 3 Division of Pediatric Surgery, University Hospital Cologne, Kerpenerstr. 62, 50937 Köln, Germany; 4 Department of Pediatric Critical Care Medicine and Neonatology, University Hospital Cologne, Kerpenerstr. 62, 50937 Köln, Germany; 5 Department of General, Visceral, Cancer and Transplantation Surgery, University Hospital Cologne, Kerpenerstr. 62, 50937 Köln, Germany; 6 Department of General, Visceral, Cancer and Transplantation Surgery, University Hospital Cologne, Kerpenerstr. 62, 50937 Cologne, Germany; 7 Department of General, Visceral and Thoracic Surgery Kreiskrankenhaus Gummersbach, Klinikum Oberberg GmbH, Wilhelm-Breckow-Allee 20, 51643 Gummersbach, Germany; 8 Department of Pediatric Surgery, University Hospital of Marburg, 35043 Baldingerstraße, Marburg, Germany

**Keywords:** Total hepatic vascular exclusion, Retrohepatic inferior vena cava injury, Juxtahepatic venous injury, Liver trauma, Transdiaphragmatic intrapericardial inferior vena cava control, Cadaveric surgical training

## Abstract

**Purpose:**

Retrohepatic inferior vena cava (IVC) injury is a rare but frequently fatal challenge in trauma surgery. This study describes a structured cadaveric training sequence for total hepatic vascular exclusion (THVE), including transdiaphragmatic intrapericardial IVC exposure as an alternative escalation route for suprahepatic venous control.

**Methods:**

A structured cadaveric dissection study was performed on nine adult cadavers by a multidisciplinary team of vascular and pediatric surgeons. The motivation was derived from an index pediatric blunt trauma case involving life-threatening retrohepatic caval bleeding. Five anatomical exposure steps relevant to THVE were rehearsed sequentially: hepatoduodenal ligament control, infrahepatic IVC exposure above the renal veins, access to the suprahepatic IVC via infradiaphragmatic and transdiaphragmatic intrapericardial IVC exposure and optional supracoeliac aortic exposure. The exposure steps were performed in a fixed sequence that was identical in all nine donors; the sequence was not randomized. After each dissection, procedural difficulty for each anatomical landmark was graded using a five-point scale: 1 = very easy, 2 = easy, 3 = moderate, 4 = difficult, and 5 = very difficult.

**Results:**

Hepatoduodenal ligament control received the lowest difficulty ratings. Perceived difficulty of infrahepatic IVC and supracoeliac aortic exposure decreased after repeated dissections. Infradiaphragmatic suprahepatic IVC exposure remained technically demanding, whereas transdiaphragmatic intrapericardial IVC exposure received significantly lower ratings of perceived difficulty (*p* = .004), with lower ratings after the initial dissections.

**Conclusion:**

In this cadaveric training model, transdiaphragmatic intrapericardial IVC exposure received significantly lower ratings of perceived difficulty than infradiaphragmatic suprahepatic IVC exposure. Because the order of the two approaches was fixed and difficulty was assessed by team consensus, this finding does not establish that the transdiaphragmatic route is objectively easier, safer, or clinically superior.

## Introduction

The present study was prompted by the case of a 7-year-old boy who sustained blunt abdominal crush trauma and presented to a district hospital in hemorrhagic shock. Emergency laparotomy revealed massive venous bleeding from a liver laceration involving segments II–III. Perihepatic packing, manual compression, the Pringle maneuver, and infrahepatic IVC control failed to control the hemorrhage, raising suspicion of hepatic venous outflow or retrohepatic caval injury.

Conventional abdominal suprahepatic caval control for total hepatic vascular exclusion was not feasible because of a rapidly expanding retroperitoneal hematoma during ongoing exsanguination. After coordinated resuscitative preparation with the anesthesiology team, the diaphragm and pericardium were opened and the intrapericardial IVC was clamped, allowing total hepatic vascular exclusion and controlled repair of the retrohepatic caval injury.

The child survived without further surgical intervention. This case provided the clinical rationale for development of the present structured cadaveric training sequence.

Management of juxtahepatic venous injuries is difficult not only because of hemorrhage severity, but also because of the complex anatomy. The retrohepatic inferior vena cava (IVC) is fixed behind the liver, embedded within the bare area, and only partially accessible during uncontrolled bleeding. The surrounding liver, diaphragm, and suspensory attachments may provide temporary tamponade; uncontrolled mobilization can release this containment and convert a partially contained bleeding into catastrophic exsanguination [1–5].

The operative response to exsanguinating liver trauma in a hemodynamically unstable patient must be sequential. Manual compression of the liver, approximation of the injured hepatic surfaces and perihepatic packing are used initially to restore hepatic contour, compress bleeding surfaces, and gain time for resuscitation [5, 6]. If packing alone is insufficient, the Pringle maneuver occludes portal triad inflow and provides both therapeutic hemorrhage control and diagnostic information regarding the source of bleeding. Persistent hemorrhage despite adequate packing and a correctly performed Pringle maneuver should therefore raise suspicion of hepatic venous outflow or retrohepatic caval injury and should prompt rapid escalation to suprahepatic and infrahepatic caval control [2, 5, 7–10].

THVE [11] is one of the few operative strategies capable of providing temporary vascular control in otherwise uncontrollable hepatic venous outflow or retrohepatic caval bleeding. It requires simultaneous control of the hepatoduodenal ligament, the infrahepatic and suprahepatic IVC. The maneuver is technically unforgiving and physiologically hazardous, because interruption of caval venous return may cause abrupt preload loss and severe hypotension. Its execution therefore depends not only on surgical exposure, but also on anesthetic-surgical coordination, timing, and immediate resuscitative preparation [8, 12–14]. When abdominal suprahepatic caval control is not feasible, transdiaphragmatic intrapericardial IVC exposure may provide a direct route for suprahepatic caval control. However, this approach requires precise anatomical orientation and prior technical familiarity [15–17].

The surgeon who performed the index operation had prior experimental liver transplantation training in canine and porcine models [18]. This likely provided uncommon familiarity with hepatic vascular exclusion. The case therefore illustrates a broader problem: rare technical competence in juxtahepatic venous trauma cannot be improvised during exsanguination; it must be rehearsed before the emergency occurs. Based on this clinical trigger and the recognized training gap in complex hepatic vascular control, we developed a structured cadaveric training sequence for surgeons without routine transplant or complex hepatobiliary vascular exposure. The aim of this study was to evaluate the feasibility and perceived difficulty of sequential anatomical exposures required for THVE in a cadaveric laboratory model. This model was intended to improve anatomical orientation and confidence with basic and advanced abdominal vascular-control maneuvers in a controlled environment.

## Methods

### Study design and ethical approval

A structured cadaveric dissection study was performed between 2024 and 2026 to evaluate the feasibility and perceived difficulty of anatomical exposures required for THVE. The study served as a translational training model derived from an index pediatric blunt trauma patient with life-threatening retrohepatic caval bleeding. The study protocol was approved by the Institutional Ethics Commission [reference number: 18 − 012; approval date: April 11, 2024]. The study was conducted in accordance with the ethical principles of the Declaration of Helsinki and applicable institutional and national regulations governing the use of donated human bodies for medical research and education. All cadaveric specimens were obtained through the Department of Anatomy’s body donation program at the University of Cologne. During their lifetime, donors provided written informed consent for the use of their bodies in medical research and education. No living human participants were involved, and no minors were included among the cadaveric donors.

### Cadaveric specimens and surgical team

Dissections were performed on eight formalin-fixed adult cadavers and one fresh-frozen adult cadaver by a multidisciplinary team of vascular and pediatric surgeons, with assistance from medical students.

### Training concept and operative sequence

**Fig. 1:**
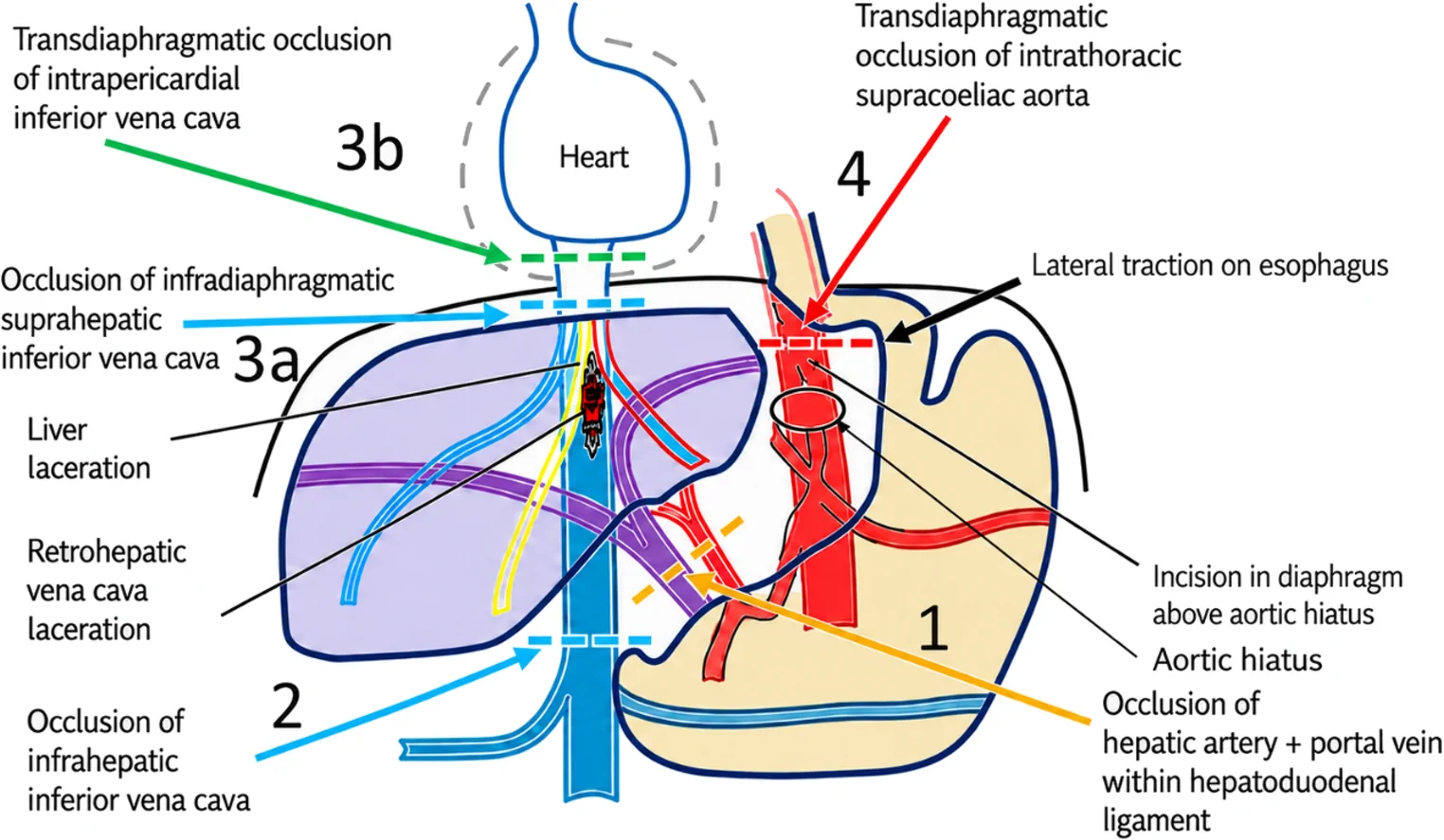
Schematic overview of the vascular-control sites required for total hepatic vascular exclusion in suspected retrohepatic IVC injury

The training sequence was based on the operative algorithm required when perihepatic packing and the Pringle maneuver fail to control torrential hepatic hemorrhage, raising suspicion of hepatic venous outflow or retrohepatic caval injury. The cadaveric exercises were designed to rehearse the required anatomical steps (Fig. 1) for step-by-step escalation from standard damage-control maneuvers to THVE.

The exposure steps were performed in a fixed sequence that was identical in all nine donors; the sequence was not randomized. The two suprahepatic approaches were performed in the same donor and in the same order, with infradiaphragmatic suprahepatic IVC exposure performed first, followed by transdiaphragmatic intrapericardial exposure.

The following exposure steps were rehearsed sequentially in each cadaver:

1. hepatoduodenal ligament control (Pringle maneuver);
2. infrahepatic IVC exposure above the renal veins;
3. infradiaphragmatic access to the suprahepatic IVC.
4. transdiaphragmatic intrapericardial access to the suprahepatic IVC.
5. supracoeliac aortic exposure;

The figure shows hepatoduodenal ligament control, infrahepatic IVC control above the renal veins, infradiaphragmatic and alternative transdiaphragmatic routes for suprahepatic caval control, and optional supracoeliac aortic control as an adjunct in profound hemodynamic instability.

### Surgical techniques

#### Hepatoduodenal ligament control

**Fig. 2:**
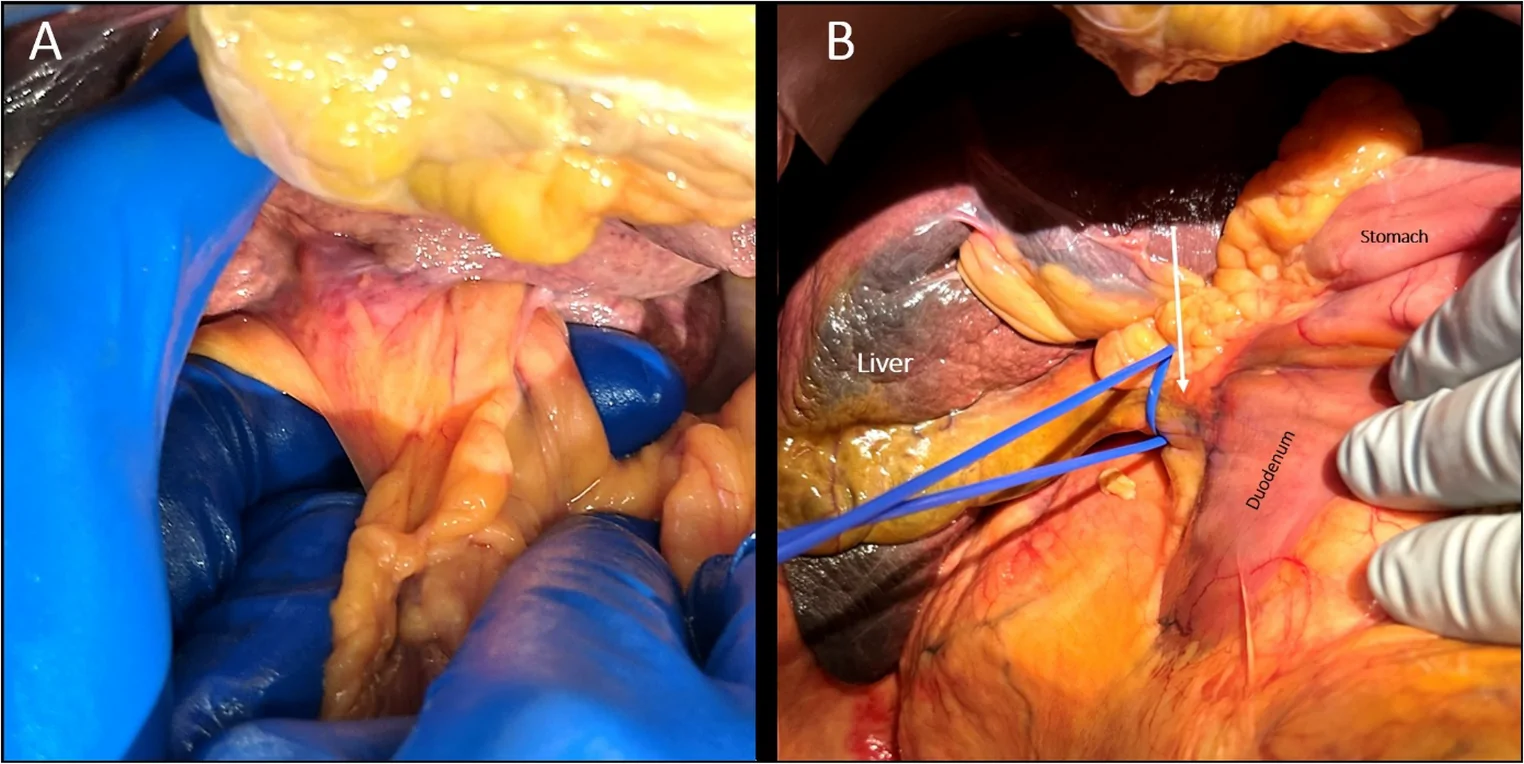
Cadaveric dissection demonstrating hepatoduodenal ligament control. (**A**) Digital passage through the foramen of Winslow behind the hepatoduodenal ligament, with the fingertip exiting through the incised hepatogastric ligament. (**B**) Vessel-loop encirclement of the hepatoduodenal ligament to simulate the Pringle maneuver

The hepatoduodenal ligament was approached through the foramen of Winslow. The surgeon’s index finger was introduced behind the hepatoduodenal ligament and a curved clamp was advanced over the fingertip to create a controlled passage around the portal triad (Fig. 2). A vessel loop or tourniquet was passed around the hepatoduodenal ligament and tightened to perform occlusion (Pringle maneuver). The endpoint of this step was reproducible encirclement and occlusion of the hepatoduodenal ligament.

#### Infrahepatic IVC exposure above the renal veins

**Fig. 3:**
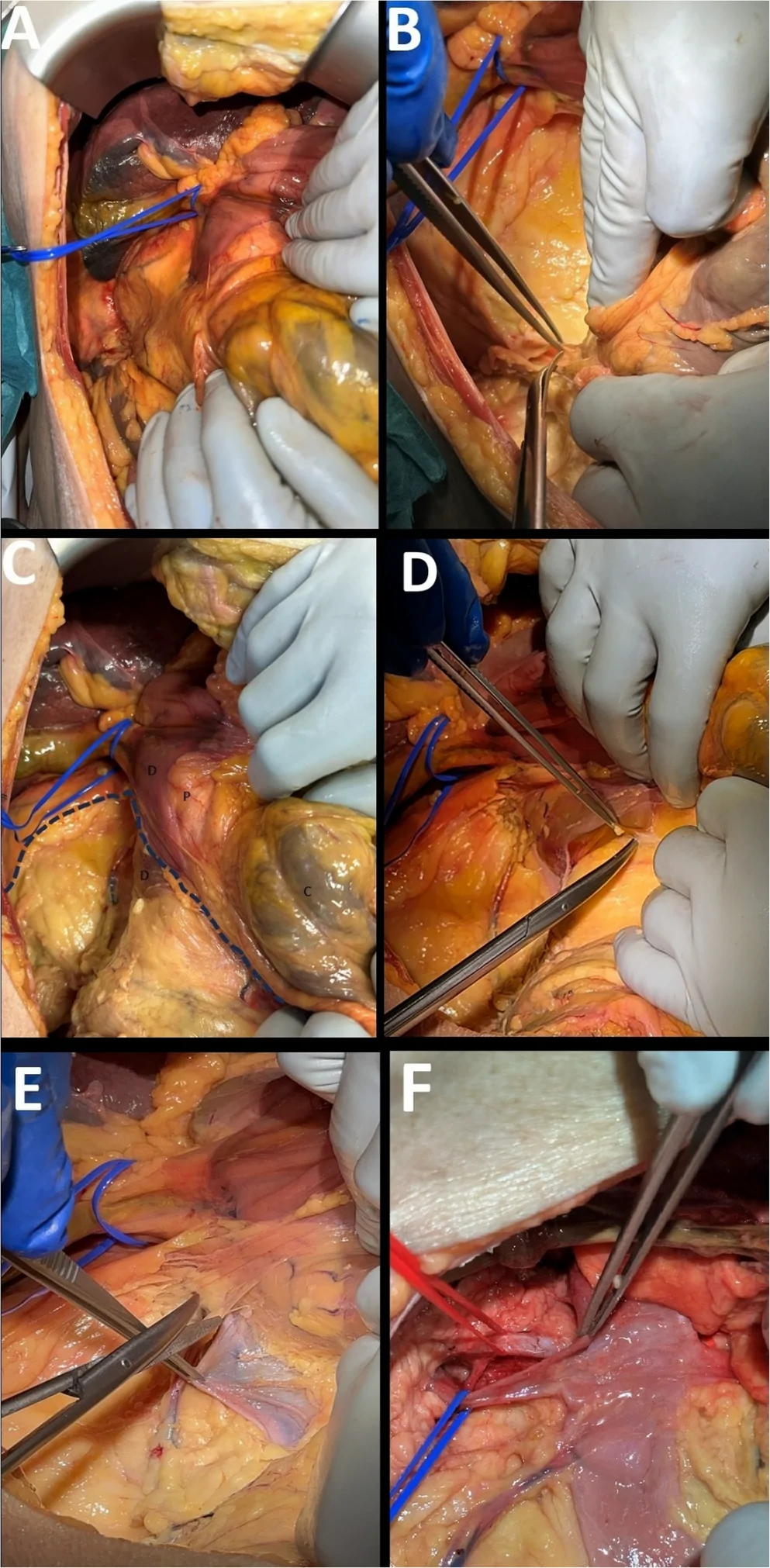
Stepwise cadaveric exposure of the infrahepatic IVC above the renal veins. (**A**) Initial right upper abdominal situs. (**B**, **C**) Medial mobilization of the right colic flexure with incision of the lateral peritoneal reflection; the dashed line marks the opened retroperitoneal plane. (**D**) Kocher mobilization of the duodenum and pancreatic head. (**E**) Progressive skeletonization of the infrahepatic IVC. (**F**) Final exposure of the infrahepatic IVC above the renal veins, with vessel loops around the right renal vein (blue) and right renal artery (red)

 The right colic flexure was mobilized medially. The peritoneal attachments along the right lateral border of the duodenum were incised, and the duodenum and pancreatic head were mobilized medially using the Kocher maneuver. This exposed the infrahepatic IVC above the renal veins. The vessel was identified and encircled with a tourniquet to simulate infrahepatic caval control. The endpoint was safe exposure and encirclement of the infrahepatic IVC above the renal veins (Fig. 3).

#### Infradiaphragmatic access to the suprahepatic IVC

**Fig. 4:**
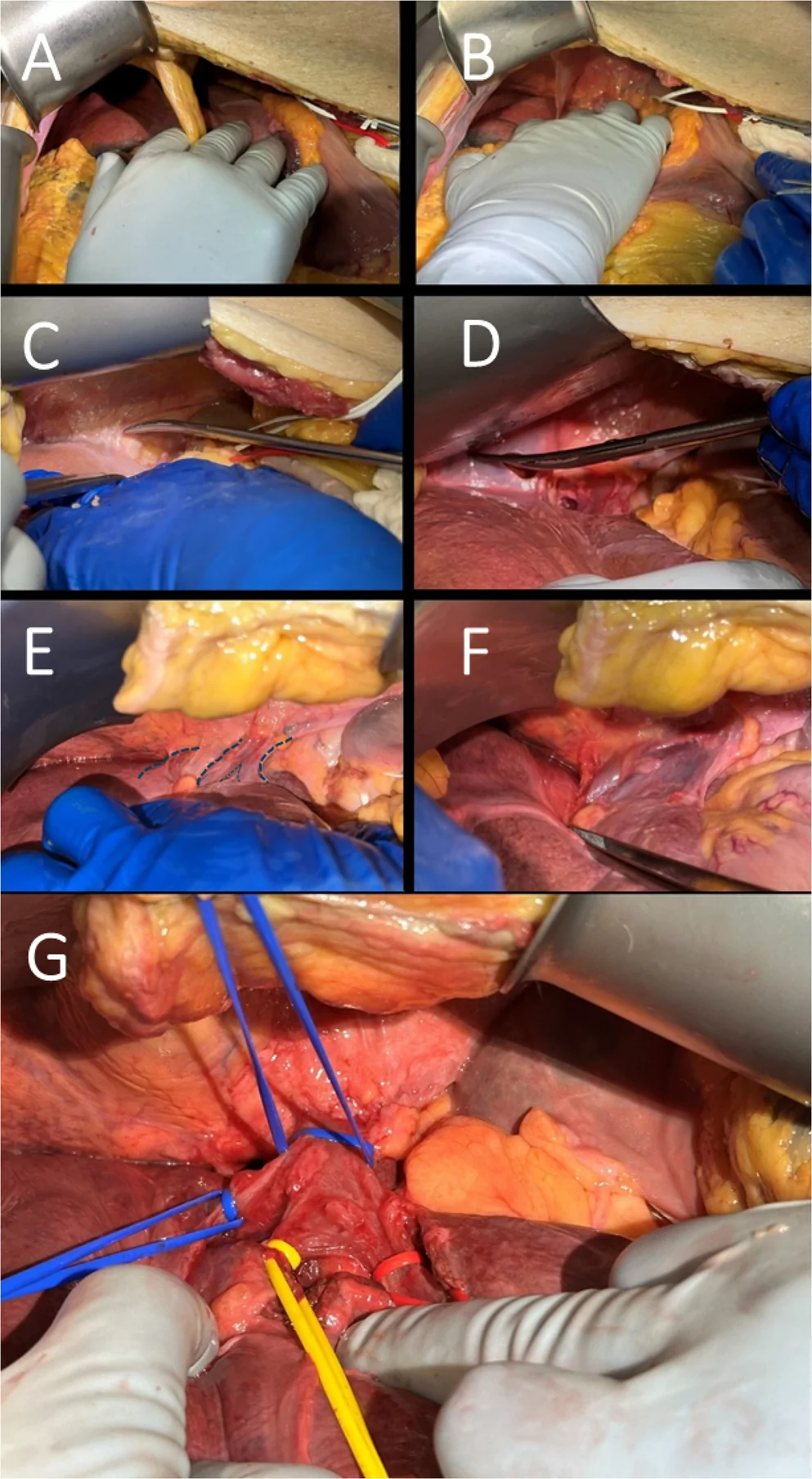
Cadaveric infradiaphragmatic exposure of the suprahepatic IVC and hepatic venous confluence. Division of the round (**A**) and falciform (**B**) ligaments. Division of the left (**C**) and right (**D**) triangular and coronary ligaments. (**E**) Identification of the right, middle, and left hepatic veins, with a common middle-left hepatic venous confluence. (**F**) Further skeletonization of the hepatic venous confluence. (**G**) Final exposure with vessel-loop control of the suprahepatic IVC below the diaphragm and above the hepatic venous confluence. The right, middle, and left hepatic veins are encircled with vessel loops

Access to the suprahepatic IVC without diaphragmatic incision, as commonly performed during liver transplantation and selected hepatic tumor resections, was rehearsed as an alternative route for suprahepatic IVC control. The liver was retracted caudally, and the falciform and coronary ligament attachments were divided as required to expose the suprahepatic IVC below the diaphragm. The hepatic venous confluence and the short extrahepatic segment of the suprahepatic IVC were identified. A tourniquet was then applied around the subdiaphragmatic suprahepatic IVC (Fig. 4).

The endpoint of this step was to determine whether safe suprahepatic IVC control could be achieved without opening the diaphragm.

#### Transdiaphragmatic intrapericardial IVC exposure

The transdiaphragmatic intrapericardial exposure was rehearsed as an alternative abdominal route for suprahepatic IVC control. In the training sequence, the round, falciform and coronary ligaments had already been divided during the preceding infradiaphragmatic exposure (step 3a), so that the liver could be retracted caudally directly. When the transdiaphragmatic route is used as the primary approach, the round and falciform ligaments are divided and the anterior coronary ligament is opened only as far as necessary to visualize the inferior surface of the anterior diaphragm; further hepatic mobilization is not required.

**Fig. 5:**
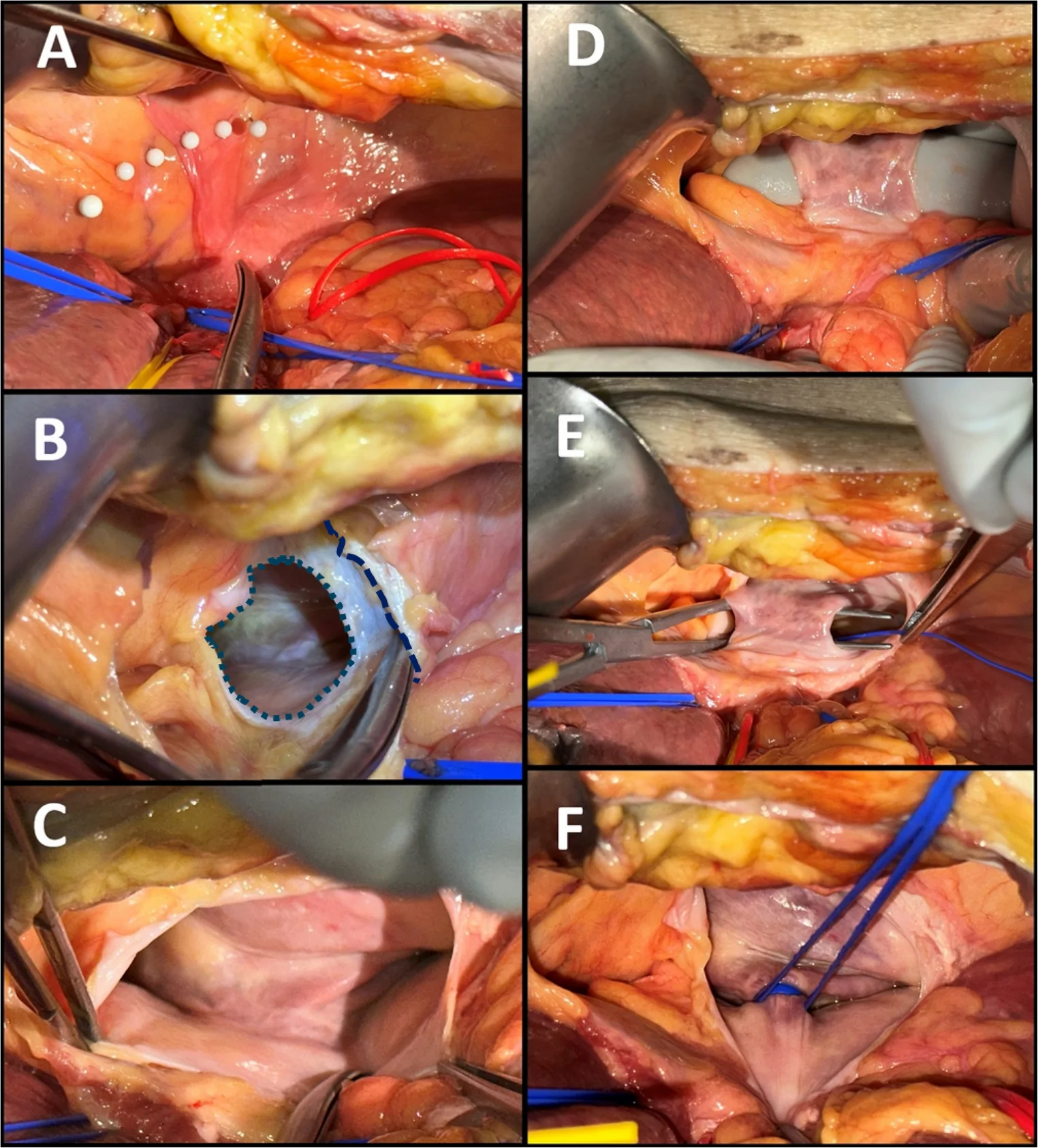
Cadaveric transdiaphragmatic intrapericardial IVC exposure. (**A**) Pins mark the planned diaphragmatic incision; vessel loops around the right, middle, and left hepatic veins. The tip of the scissors marks the suprahepatic IVC. (**B**) Opened diaphragm (dashed line) indicating the diaphragmatic and pericardial edges (dashed circle). (**C**) Intrapericardial exposure of the IVC beneath the heart. (**D**, **E**) Digital and instrumental passage behind the intrapericardial IVC. (**F**) Final vessel-loop control of the intrapericardial IVC for simulated suprahepatic caval occlusion

A curved transverse diaphragmatic incision was made approximately 2 cm anterior to the suprahepatic IVC, which in the training sequence had already been exposed from below. The incision was continued through the underlying pericardium. The intrapericardial segment of the IVC was identified. A plane behind the IVC was developed bluntly with the surgeon’s index finger, and a tourniquet was passed around the intrapericardial IVC (Fig. 5). The endpoint was reproducible identification of the transdiaphragmatic intrapericardial window and safe encirclement of the intrapericardial IVC for suprahepatic caval control.

#### Supracoeliac aortic exposure

Supracoeliac aortic exposure was rehearsed as an optional adjunct for proximal arterial control in profound hemodynamic instability. The lesser sac was opened longitudinally, with care taken to avoid injury in the presence of variant hepatic arterial anatomy. The intra-abdominal esophagus was identified, mobilized, encircled with a vessel loop, and retracted to the left. Dissection was then directed posterior to the esophagus, toward the aorta. In this cadaveric model with an absent aortic pulse, the dissection relied on palpation of the aortic wall against the vertebral column. 

**Fig. 6:**
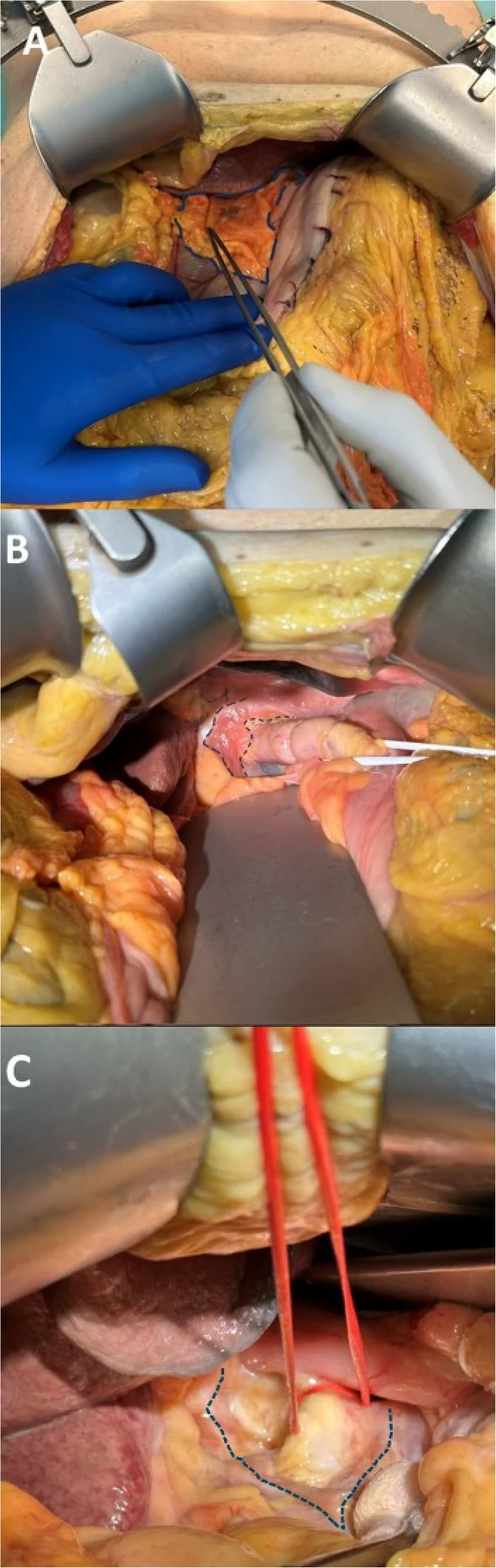
Cadaveric supracoeliac aortic exposure. (**A**) The dashed line marks the hepatogastric ligament before entry into the lesser sac. (**B**) After incision of the hepatogastric ligament and opening of the peritoneal covering over the distal esophagus, the esophagus is retracted laterally; the dashed line indicates the right diaphragmatic crus. (**C**) Following longitudinal incision of the right diaphragmatic crus (dashed lines), the supracoeliac aorta is exposed and encircled with a vessel loop for illustration

The right diaphragmatic crus was incised longitudinally, and blunt dissection was carried out along the anterior aortic wall, down to the vertebral column. A clamp was then positioned with its tips oriented toward the vertebral column. The endpoint was identification and exposure of the supracoeliac aorta for potential clamping (Fig. 6).

### Difficulty assessment and statistical analysis

After each dissection, the surgical team jointly assessed the difficulty of each anatomical exposure using a five-point Likert-type ordinal scale. A score of 1 indicated very easy exposure (readily identifiable, with few easily dissected structures); 2 reflected easy exposure (readily identifiable landmarks but a greater number of structures requiring straightforward dissection); 3 indicated moderate difficulty (limited visibility requiring extensive dissection of multiple landmarks prior to identification); 4 denoted difficult exposure (impaired visibility during extensive dissection); and 5 represented very difficult exposure (similar to 4, but associated with a high risk of iatrogenic injury). The score reflected anatomical accessibility, clarity of landmarks, working space, extent of dissection required, perceived risk of iatrogenic injury, and reproducibility of the exposure.

Descriptive analysis detailed the distribution of difficulty scores and their medians. Paired comparison of transdiaphragmatic intrapericardial IVC exposure and infradiaphragmatic suprahepatic IVC exposure was performed using the Wilcoxon signed-rank test. A p value < 0.05 was considered statistically significant.

## Results

### Hepatoduodenal ligament control

**Fig. 7:**
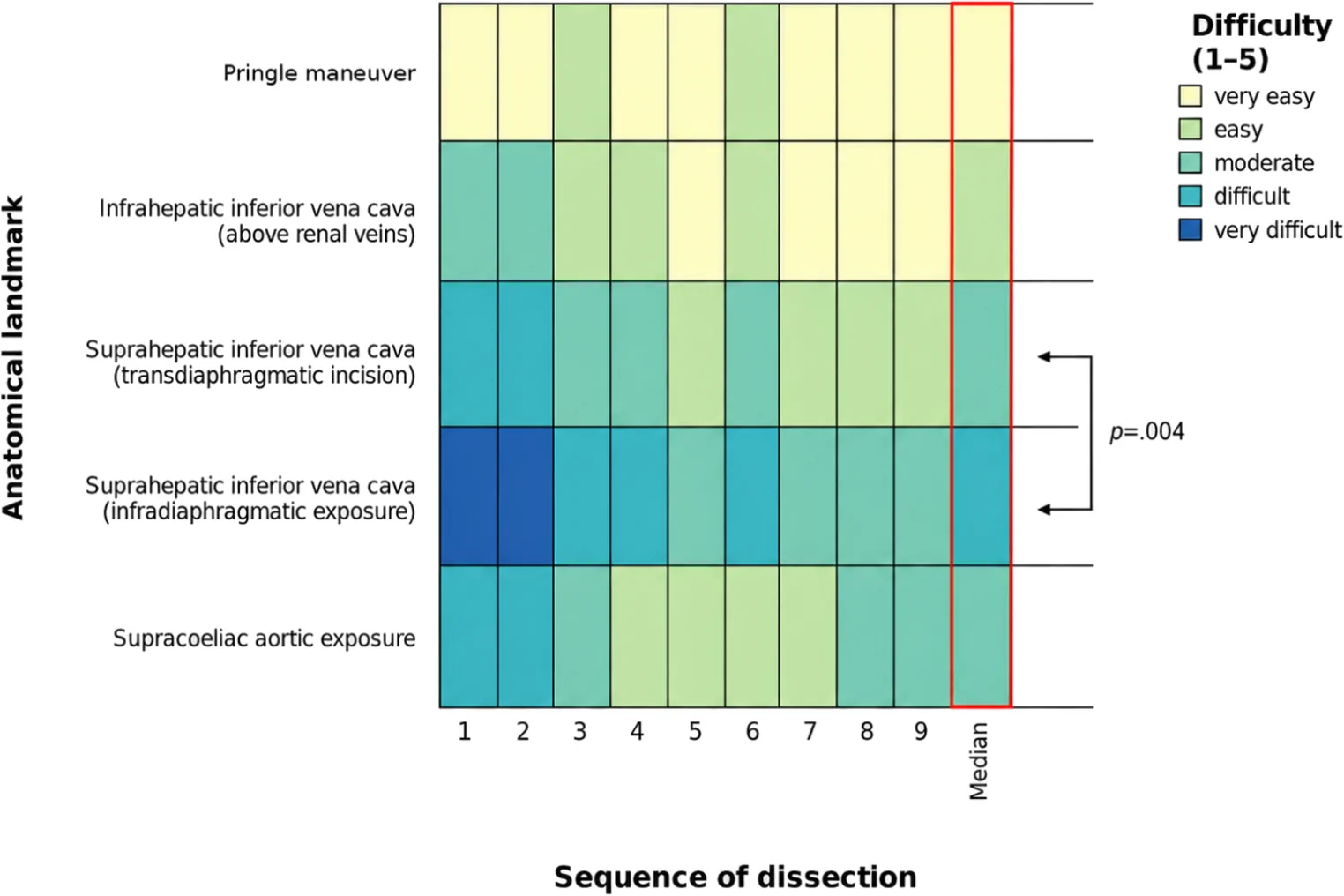
Heatmap showing perceived difficulty of the anatomical exposure steps across sequential cadaveric dissections. Difficulty was graded on a five-point ordinal scale from 1 = very easy to 5 = very difficult. The final column shows the median rating for each step. Infradiaphragmatic suprahepatic IVC exposure received significantly higher perceived-difficulty ratings than transdiaphragmatic intrapericardial IVC exposure (*p* =.004)

Hepatoduodenal ligament control was the least difficult and most consistently reproducible maneuver (Fig. 7). Occlusion of the hepatoduodenal ligament (Pringle maneuver) was rated as very easy in seven cadavers and easy in two cadavers, corresponding to a median difficulty score of 1 (very easy; range 1–2). An aberrant right hepatic artery arising from the superior mesenteric artery was identified during subsequent dissection in one cadaver; however, this did not prevent effective tourniquet control of the hepatoduodenal ligament.

### Infrahepatic IVC exposure above the renal veins

Exposure of the infrahepatic IVC above the renal veins was rated as moderate in the first two cadavers and as easy or very easy in the subsequent seven cadavers. The median difficulty score was 2 (easy; range 1–3).

### Suprahepatic IVC exposure

The two approaches to suprahepatic IVC control showed different difficulty profiles. Transdiaphragmatic intrapericardial IVC exposure was rated as difficult in the first two cadavers, reflecting the initial learning curve required to identify the optimal diaphragmatic incision site and to develop familiarity with blunt dissection behind the intrapericardial IVC. In subsequent dissections, difficulty decreased to moderate or easy. The median difficulty score was 3 (moderate; range 2–4).

In contrast, infradiaphragmatic access to the suprahepatic IVC remained technically demanding throughout the series. It was rated as very difficult in the first two cadavers, primarily because of the short subdiaphragmatic segment of the suprahepatic IVC and the restricted operative space above the hepatic venous confluence. These limitations would likely be amplified in the clinical setting by retrohepatic hematoma and active venous bleeding. In all subsequent dissections, infradiaphragmatic exposure was rated as difficult or moderate; no infradiaphragmatic suprahepatic IVC exposure was rated as easy or very easy. The median difficulty score was 4 (difficult; range 3–5).

In comparison, infradiaphragmatic suprahepatic IVC exposure received significantly higher perceived-difficulty ratings than transdiaphragmatic intrapericardial IVC exposure (*p* =.004) (Fig. 7). In our experience, the principal limitation of the infradiaphragmatic approach was the restricted operative space above the hepatic venous confluence, largely due to the short infradiaphragmatic segment of the suprahepatic IVC. By contrast, the transdiaphragmatic intrapericardial route avoided extensive subdiaphragmatic dissection in the region where hematoma and active bleeding would be expected clinically. It also received lower perceived-difficulty ratings while allowing exposure of the suprahepatic IVC without manipulation in close proximity to the hepatic veins.

### Supracoeliac aortic exposure

Supracoeliac aortic exposure was rated as difficult in the first two cadavers, partially because the absence of aortic pulsation made anatomical orientation more challenging. With repeated dissection, familiarity with identifying the aorta despite the absence of pulsation increased. Consequently, perceived difficulty decreased in the subsequent cadavers, with ratings of moderate or easy. The median difficulty score was 3 (moderate; range 2–4). 

## Discussion

This cadaveric study was designed to translate a rare pediatric survival scenario into a structured anatomical training sequence for total hepatic vascular exclusion (THVE). All predefined vascular-control steps could be rehearsed in the cadaveric laboratory. Hepatoduodenal ligament control was consistently the least difficult maneuver, whereas infrahepatic IVC and supracoeliac aortic exposure were associated with lower perceived-difficulty ratings after repeated dissections. The most relevant finding concerned suprahepatic caval control: infradiaphragmatic exposure remained technically demanding throughout the series, whereas transdiaphragmatic intrapericardial exposure received significantly lower consensus ratings of perceived difficulty, particularly after the initial dissections. Because the two approaches were performed in a fixed sequence and difficulty was assessed by team consensus, this finding should be interpreted as an observation within a structured training model rather than as evidence of clinical superiority.

In hemodynamically stable patients, blunt hepatic injuries are predominantly managed non-operatively, and selected penetrating injuries may also be managed conservatively in appropriate settings [7, 8, 19]. Reed et al. reported that approximately 85–90% of adult blunt liver injuries are currently managed non-operatively [8], while a recent multicenter pediatric cohort reported a non-operative management failure rate of only 1% [20]. These data define the contemporary treatment paradigm: most hepatic injuries no longer require operative intervention, whereas unstable non-responders with ongoing hemorrhage represent a rare but critical exception.

This shift toward non-operative management creates a parallel educational problem. Operative exposure to complex hepatic hemostatic maneuvers during surgical training has become increasingly limited. Lucas and Ledgerwood reported that residents encounter operative hemostatic maneuvers for liver injury only rarely during training, and surveys of general surgery trainees demonstrate limited confidence in complex hepatopancreatobiliary procedures [21, 22]. Elective experience with THVE is concentrated predominantly within specialized hepatobiliary and transplant practice [6, 23–26].

Consequently, the surgeon confronted unexpectedly with catastrophic juxtahepatic venous injury may have little or no prior practical experience with the required sequence of vascular-control maneuvers.

In an unstable patient with exsanguinating hepatic trauma, operative management remains mandatory [7, 8]. The first operative response to exsanguinating hepatic trauma is sequential: manual compression, restoration of hepatic contour, perihepatic packing [9], and then Pringle maneuver if bleeding persists. Perihepatic packing remains one of the most widely used and effective damage-control maneuvers for severe liver trauma [9, 10]. The timing of pack removal remains debated, with different series reporting complications and rebleeding risks after early or delayed removal [27–29]. Interposition of a non-adherent plastic drape between the liver and laparotomy pads may reduce adhesion to the hepatic surface and disruption of the fragile hemostatic clot during subsequent pack removal [30].

The index case illustrates the diagnostic importance of continued venous hemorrhage despite adequate packing and Pringle occlusion. The Pringle maneuver interrupts portal triad inflow and therefore reduces bleeding from hepatic arterial and portal venous sources. Persistent venous hemorrhage despite adequate packing and Pringle occlusion should consequently raise immediate concern for hepatic venous outflow or retrohepatic caval injury [2, 5, 8]. This distinction was central to the management of the child: repeated packing could not definitively control a retrohepatic caval source, whereas uncontrolled hepatic mobilization before vascular control risked releasing the temporary tamponade surrounding the injured retrohepatic IVC.

Juxtahepatic venous injuries remain particularly difficult because the severity of hemorrhage is compounded by complex anatomy. The retrohepatic IVC is fixed behind the liver within the bare area and is closely related to the hepatic venous confluence, diaphragm, suspensory ligaments, and multiple short hepatic veins draining directly into the cava. These structures may temporarily contain hemorrhage; disruption of this containment before vascular control can precipitate catastrophic exsanguination [1–5]. Buckman et al. [2] described three principal operative strategies: direct repair with or without vascular isolation, hepatic resection when primary repair is not otherwise feasible, and strategic tamponade as part of damage-control surgery.

Infrahepatic IVC control is an essential component of THVE but cannot control hepatic venous outflow in isolation. In the index operation, right medial visceral rotation and Kocher mobilization enabled control of the infrahepatic IVC above the renal veins, yet hemorrhage persisted. In the cadaveric model, perceived difficulty of this exposure decreased with repeated dissection as the team became more familiar with the right colic flexure, duodenum, pancreatic head, renal venous landmarks, and retroperitoneal plane. Persistent hemorrhage despite infrahepatic control in the clinical case nevertheless emphasized the requirement for suprahepatic control above the hepatic venous confluence.

The critical technical decision in the index operation was to abandon conventional infradiaphragmatic suprahepatic exposure when torrential hemorrhage and the expanding retroperitoneal hematoma made this route impractical. Transdiaphragmatic intrapericardial IVC control was used instead. In the cadaveric model, infradiaphragmatic exposure remained demanding because of the restricted working space, proximity of the hepatic venous confluence, and the short extrahepatic segment of the suprahepatic IVC. These anatomical constraints are particularly relevant in a field further compromised by hematoma and active venous hemorrhage.

The findings concerning the two suprahepatic routes should therefore be considered at three levels. The observed result was that transdiaphragmatic intrapericardial exposure received significantly lower consensus ratings of perceived difficulty than infradiaphragmatic exposure in this sequential cadaveric model. Anatomically, the larger working space above the diaphragm and avoidance of dissection immediately adjacent to the hepatic venous confluence provide plausible explanations for this difference. However, the study cannot establish that the transdiaphragmatic approach is objectively easier, safer, or clinically superior. Its practical implication is more limited: the transdiaphragmatic route can be rehearsed as an escalation maneuver for situations in which conventional infradiaphragmatic suprahepatic control is not feasible or would require hazardous dissection close to the hepatic venous confluence.

This interpretation is consistent with previous anatomical and operative reports.

Shchukin et al. [15] evaluated transabdominal approaches to the supradiaphragmatic IVC and right atrium and reported that diaphragmotomy provided a direct route to this region. Tohyama et al. [16] and Miyazaki et al. [31] described intrapericardial IVC exposure during hepatectomy with THVE and emphasized the advantage of obtaining suprahepatic caval control while avoiding extensive dissection around the hepatic venous confluence. Pediatric trauma reports have likewise demonstrated that supradiaphragmatic caval control can be feasible in selected catastrophic retrohepatic venous injuries [32, 33].

The intrapericardial approach should nevertheless be regarded as an escalation maneuver rather than a routine exposure. It requires accurate localization of the diaphragmatic and pericardial window, avoidance of cardiac and phrenic nerve injury, and precise orientation to the intrapericardial IVC and right atrium. In the cadaveric training sequence, the diaphragmatic incision was positioned approximately 2 cm anterior to a suprahepatic IVC that had already been exposed during the preceding infradiaphragmatic dissection. This reference may not be available during an exsanguinating emergency, when hematoma and hemorrhage obscure the suprahepatic cava. Indeed, in the index operation the diaphragm and pericardium were opened without previous infradiaphragmatic exposure. Emergency translation therefore requires familiarity with anatomical landmarks that remain recognizable despite loss of direct caval visualization. Tohyama et al. [16] described a landmark-based transmediastinal intrapericardial approach, and incorporation of such landmark-based orientation represents an important refinement for future training.

The sequential difficulty ratings suggested a steep early learning curve in perceived difficulty, particularly for the transdiaphragmatic approach. During the first two cadaveric dissections, the principal challenges were identification of the diaphragmatic entry site and development of confidence with blunt dissection along the dorsal aspect of the intrapericardial IVC. Thereafter, this steep initial reduction was followed by a relative plateau, with consistently lower perceived-difficulty ratings. We attributed this pattern primarily to increasing familiarity with the working space above the diaphragm and with circumferential caval control away from the hepatic venous confluence. A learning effect was also observed for the infradiaphragmatic route, although identification and safe encirclement of the short suprahepatic caval segment remained challenging despite repeated practice.

Based on our experience, approximately six cadaveric dissections may provide sufficient repetition for the team to become familiar with the operative sequence in a training setting. This number should be regarded strictly as an experience-based estimate rather than as a validated threshold for clinical competency. The present scores reflect team-level perceived difficulty rather than the performance of individual surgeons. Competency for independent emergency use should ultimately be defined by repeated successful performance against objective criteria rather than by a fixed number of cadaveric specimens.

The availability of hepatobiliary or liver transplant expertise may be particularly valuable in catastrophic juxtahepatic venous injury because of prior experience with hepatic mobilization, THVE, major hepatic resection, and vascular control around the hepatic venous confluence. This familiarity may facilitate earlier recognition of the likely source of persistent venous hemorrhage and more precise judgment regarding whether escalation to total hepatic vascular exclusion is warranted. This decision is particularly important because THVE is a high-risk maneuver with major physiological consequences and should not be undertaken indiscriminately. When THVE is required, such expertise may facilitate controlled vascular isolation, caval repair, and any necessary hepatic resection while minimizing ongoing blood loss and hepatic ischemia.

However, catastrophic juxtahepatic venous injury may initially present in a hospital without immediate hepatobiliary or transplant support, as occurred in the index case. The educational objective should therefore not be to confine these maneuvers to hepatobiliary specialists, but to ensure that surgeons responsible for emergency laparotomy can recognize the injury pattern, initiate appropriate damage-control measures, and understand the available rescue options while specialist expertise is activated as early as possible.

Management is inherently multidisciplinary. Close coordination with anesthesiology is essential because suprahepatic and infrahepatic caval occlusion can abruptly reduce venous return, lower preload, aggravate hypotension, and precipitate circulatory collapse [12–14].

In the index case, caval exposure was deliberately not followed by immediate clamping; manual compression and approximation of the liver were maintained while the anesthesiology team prepared the child for interruption of venous return through rapid transfusion and volume resuscitation. Vascular surgical expertise may be required for complex caval repair or reconstruction, and cardiothoracic assistance may become necessary if the injury or exposure extends toward the intrapericardial IVC or right atrium. Interventional radiology may contribute after initial hemorrhage control and physiological stabilization, particularly in the management of persistent or recurrent arterial bleeding. Predefined multidisciplinary pathways that facilitate rapid access to these specialties are therefore an important component of trauma-system preparedness.

Supracoeliac aortic exposure was included in the training sequence as an optional rescue maneuver rather than a routine component of THVE [14, 34, 35]. In the cadaveric model, the absence of aortic pulsation made orientation dependent on palpation of the vessel against the vertebral column. Perceived difficulty decreased with repeated dissection. Supracoeliac control may therefore represent a useful bailout adjunct in selected circumstances, but it does not directly control retrohepatic venous hemorrhage and does not replace suprahepatic and infrahepatic venous isolation.

Alternative strategies for retrohepatic IVC and hepatic venous injuries have been described, but each has important limitations. Venovenous bypass has been used successfully in selected institutions and may mitigate some of the hemodynamic consequences of caval occlusion [36, 37], but it is rarely immediately available during emergency laparotomy, particularly in district hospitals or resource-limited settings. Atriocaval shunting, originally described by Schrock et al. [38] has been associated with mortality rates of approximately 70–90% [39]. Its application requires immediate equipment availability, rapid technical execution, and frequently median sternotomy, and is particularly challenging once profound blood loss, cardiac arrest, or coagulopathy has developed.

The educational implications of the present model are therefore central. The index case provided a defined clinical objective around which training could be structured. Rather than undertaking an open-ended anatomical dissection, the team rehearsed a specific emergency scenario and the sequential maneuvers required to manage it. In our experience, this goal-directed, case-based format maintained engagement and provided different educational benefits according to training level. Junior residents improved anatomical orientation, senior residents and recently board-certified surgeons rehearsed increasingly complex operative exposures, medical students consolidated applied anatomy, and experienced surgeons refined operative strategies and discussed alternative approaches. A graded team structure, with an experienced surgeon acting as mentor and progressively more advanced components assigned according to training level, therefore appears particularly suitable for this type of rehearsal.

More broadly, cadaveric and simulation studies support the value of such training for improving anatomical familiarity, technical confidence, and procedural performance, while emphasizing that these models should be regarded as educational tools rather than as evidence of improved clinical outcomes [40–46].

For the advanced components of THVE, the principal target population would be senior residents approaching independent emergency duty, fellows, and recently or experienced board-certified surgeons. The broader educational principle is relevant to surgical disciplines that operate within the abdomen, including trauma, general, pediatric, and vascular surgery. This is particularly important in hospitals without immediate hepatobiliary or transplant cover, where the surgeon performing the emergency laparotomy may initially have to recognize the injury and initiate vascular-control measures without specialist assistance. A further advantage of formalin-fixed donors is logistical flexibility. Training can be distributed over several short sessions instead of being confined to a single intensive exercise. In our program, small teams could perform approximately two-hour sessions after routine clinical duties, interrupt the dissection at any stage, and resume it on another day. Photographic and video documentation allowed anatomy, operative strategy, and technical difficulties to be reviewed between sessions before proceeding. This longitudinal format created time for reflection, repetition, and progressive participation of additional trainees. Such a structure may be transferable to other low-frequency, high-risk emergency procedures. Standardized operative sequences can be combined with institutional emergency protocols, repeated cadaveric rehearsal, and interval review of recorded material. Regional cadaver laboratories could serve as training hubs, while participating hospitals maintain local protocols defining escalation criteria, team roles, equipment, massive-transfusion preparation, and pathways for early specialist involvement. Periodic refresher sessions may be particularly important for procedures that are rarely encountered in clinical practice.

Several limitations require explicit consideration. First, difficulty was assessed by a single consensus score from the dissecting team. The raters were neither independent nor blinded and were aware of the index case in which transdiaphragmatic intrapericardial control had been decisive. Expectation bias in the direction of the observed result therefore cannot be excluded. Consensus scoring also precludes assessment of inter-rater reliability and may conceal disagreement among individual participants.

Second, the two suprahepatic approaches were performed in a fixed, non-randomized sequence. In all cadavers, the infradiaphragmatic route preceded the transdiaphragmatic exposure. This order was deliberately chosen to reproduce the clinical escalation strategy in which conventional infradiaphragmatic suprahepatic control would be attempted first and the transdiaphragmatic route used when that exposure is not feasible or would require hazardous dissection near the hepatic venous confluence. Nevertheless, the transdiaphragmatic approach was therefore always performed after hepatic mobilization and previous exposure of the suprahepatic region and may have benefited from the preceding dissection and increasing familiarity with the individual specimen. Although performing both approaches in the same donor permitted direct anatomical comparison under otherwise similar conditions, the fixed-order design cannot determine how much of the difference in perceived difficulty was attributable to anatomy and how much to carryover from the preceding exposure.

Third, no objective procedural-performance outcomes were prospectively collected. The study was designed primarily to define and illustrate the operative sequence, and dissections were repeatedly interrupted for photography, video documentation, repositioning, and discussion of anatomical landmarks. Retrospectively reconstructed procedural times would therefore not reflect technical performance. No structured technical-skills assessment was performed. Accordingly, the sequential reduction in perceived difficulty should be interpreted as increasing anatomical and procedural familiarity rather than as a formally validated individual learning curve or demonstration of competency. Future studies should prospectively assess time to successful vascular control, accuracy of encirclement or clamp placement, number of instructor prompts or interventions, predefined technical errors, and structured technical-skill scores assigned by independent observers [40, 41].

Fourth, eight of the nine donors were formalin-fixed and only one was fresh-frozen. Fixation alters tissue compliance and may influence hepatic mobilization, diaphragmatic incision, vascular exposure, and blunt development of tissue planes. Because only one fresh-frozen donor was included, and because any observation from that specimen would be confounded by its position within the sequential training series, the present study cannot determine either the magnitude or direction of the effect of preservation method.

Finally, the absence of perfusion and active hemorrhage represents the principal gap between this anatomical model and the clinical emergency. A non-perfused cadaver is well suited to initial familiarization with anatomical landmarks and operative sequence but cannot reproduce active venous hemorrhage, progressive loss of visibility, expanding hematoma, disruption of temporary tamponade, time pressure, trauma-induced coagulopathy, or the physiological consequences of caval occlusion. The present difficulty ratings therefore reflect anatomical accessibility under non-bleeding conditions and should not be assumed to represent procedural difficulty during active hemorrhage.

The model could be developed toward greater clinical fidelity in stages. Perfused fresh cadaver systems have been described for surgical simulation and trauma training [43, 44, 47]. A standardized retrohepatic or juxtahepatic caval defect could be created under perfusion so that the operative sequence is performed in the presence of ongoing simulated hemorrhage.

Despite these limitations, the present study provides a practical framework for training in a rare but catastrophic injury pattern. The central operative lesson from the index case is that persistent venous hemorrhage despite adequate packing and Pringle occlusion should prompt suspicion of hepatic venous outflow or retrohepatic caval injury and consideration of controlled venous isolation. In this cadaveric model, transdiaphragmatic intrapericardial exposure received lower consensus ratings of perceived difficulty than infradiaphragmatic suprahepatic exposure, while offering a rehearsable escalation route when conventional control is not feasible. The physiological lesson is that THVE must be synchronized with resuscitative preparation and multidisciplinary communication. The broader educational lesson is that competence in rarely performed rescue procedures cannot be improvised during exsanguination and should instead be anticipated through structured, goal-directed rehearsal.

## Conclusion

Retrohepatic caval and hepatic venous outflow injuries are rare exceptions in modern non-operative liver trauma management but remain among the most lethal patterns of hepatic injury. Persistent hemorrhage despite adequate packing and Pringle occlusion should raise immediate suspicion of juxtahepatic venous bleeding and prompt consideration of controlled venous isolation. In this cadaveric training model, transdiaphragmatic intrapericardial IVC exposure received significantly lower consensus ratings of perceived difficulty than infradiaphragmatic suprahepatic IVC exposure, with lower ratings after the initial dissections. Cadaveric rehearsal may nevertheless provide a structured means of teaching the anatomy, operative sequence, and potential danger zones of THVE to surgeons who may rarely encounter catastrophic juxtahepatic venous trauma.

## Acknowledgements

We gratefully acknowledge the donors and their families for their invaluable contribution to medical education and research, and the technical staff of the Department of Anatomy for their support during the cadaveric dissections.

## Data Availability

No datasets were generated or analysed during the current study.
